# Trematode Fluke *Procerovum* *varium* as Cause of Ocular Inflammation in Children, South India

**DOI:** 10.3201/eid2202.150051

**Published:** 2016-02

**Authors:** Lalan Kumar Arya, Sivakumar R. Rathinam, Prajna Lalitha, Usha R. Kim, Sudeep Ghatani, Veena Tandon

**Affiliations:** Aravind Medical Research Foundation, Madurai, India (L.K. Arya, P. Lalitha);; Aravind Eye Hospital and Postgraduate Institute of Ophthalmology, Madurai (S.R. Rathinam, U.R. Kim);; North-Eastern Hill University, Shillong, India (S. Ghatani, V. Tandon)

**Keywords:** granuloma, uveitis, trematode, cercaria, real-time PCR, sequencing, ribosomal DNA, eye disease, child, fresh water, lakes, ponds, rivers, parasites, India

## Abstract

Trematodes are recognized as a group of emerging parasites in tropical countries. We identified a trematode as a cause of ocular granulomas that developed in children who bathed in ponds or rivers in South India. DNA was isolated from patients’ surgically excised granulomas and from the trematode cercariae (larvae) released by the snail *Melanoides* *tuberculata* in water in which the children bathed. Real-time and conventional PCRs were performed that targeted ribosomal DNA regions spanning the internal transcribed spacer 2 and 28S sequences of this trematode. The PCR-amplified products were subjected to bidirectional sequencing. Analysis of sequences for the granuloma samples and the trematode cercariae showed maximum sequence similarity with *Procerovum* *varium* (family Heterophyidae). Our results confirmed the etiology of the ocular infection, implicating snail vectors as environmental risk factors for ocular parasitosis.

## Abstract 2

Larvae of this fluke are novel causes of granulomatous eye disease in children.

Diseases caused by helminths (e.g., nematodes, cestodes, and trematodes) are a major public health concern worldwide, particularly in developing countries because of poor hygiene, lack of public health education, and limited medical resources (*1**,**2*). The people of Southeast Asia are especially at risk for exposure to at least 70 species of foodborne and waterborne trematodes, including blood flukes, intestinal flukes, liver flukes, and lung flukes (*3*). However, epidemiologic data on parasitic diseases of trematode origin in the Indian subcontinent are scarce because of lack of screening programs. The diagnosis of parasitic diseases is much more difficult when the patient is not a definite host but is instead an intermediate or accidental host; in such cases, fecal egg identification is of no use. Serum samples are also unreliable mainly because of cross-reactive antigens (*4**,**5*). Molecular diagnostics can play a vital role in overcoming these obstacles and may lead to a precise diagnosis (*6**,**7*).

Ocular infections caused by helminths in human are rare. Among helminths that affect the eye, few have a natural predilection for the eye as their habitat; consequently, ocular invasion may occur by accident but results in eye disease (*8*). Several sporadic reports from various parts of the world have identified trematodes (i.e., *Philophthalmus* spp., *Fasciola hepatica*, and schistosomes) in the conjunctival sac and anterior chamber of the eye (*9**–**16*). On the basis of histopathologic work-up, researchers from our hospital previously reported possible trematode infection in a large group of children with ocular inflammation (*17**,**18*). We later reported molecular evidence that established the trematode *Procerovum* *varium* as the source of the ocular granuloma in a single patient from the same region as that of the children in the previous study (*19*).

Members of the genus *Procerovum* (class Trematoda, subclass Digenea, family Heterophyidae, subfamily Haplorchiinae) parasitize predominantly fish-eating birds, which are the definitive hosts. In the definitive host, the cercariae mature to adults and lay eggs that are discharged with the host feces into the environment and surrounding bodies of water. The eggs hatch to release free-swimming miracidia, which infect snails, the first intermediate hosts. The cercariae released from the snails use various freshwater and marine fish as the second intermediate hosts (*20**,**21*)*.* Humans become accidental hosts when they are exposed to these cercariae and become infected by eating infected, uncooked fish. *Procerovum* spp. are known to inhabit China, Japan, the Philippines, Australia, Taiwan, India, Vietnam, and Thailand (*20**–**24*). Ocular parasitosis caused by *Procerovum* spp. was first reported in fish in the Philippines (*22*)*.* In South India, infection with the trematode *P. varium* was reported in the pond heron *Ardeola* *grayii*, and heavy infections with metacercariae were also found in the fish *Oryzias* *melastigma* (*21*).

Identifying the exact species of trematode in granulomatous tissue is morphologically and histopathologically difficult for various reasons. First, when the granuloma is aspirated with a fine needle, the parasite comes out in pieces and loses its morphology. Second, the parasite degenerates rapidly because of an immunologic reaction in the host. Recent development of molecular methodologies like real-time PCR, sequencing, and BLAST analysis (http://blast.ncbi.nlm.nih.gov/Blast.cgi) offer opportunities for identifying the parasite at the species level (*25**–**28*).

Our previous study involving a single patient with ocular granuloma pointed to infection by the trematode *P. varium* (*19*). During March 2010–February 2013, ocular complications were being detected in children in South India in whom ocular granulomas developed after they were exposed to snail-infested water in ponds or rivers. We sought to confirm the etiology of the granulomatous eye disease. To discover and ascertain the causative agent of the disease, we performed DNA-based molecular analysis, targeting trematode larvae as they developed and were subsequently released by the vector snails.

## Materials and Methods

### Patients’ Granuloma Sample Analysis

The study protocol was approved by the Institutional Review Board of the Aravind Eye Care System, Madurai, India. All procedures adhered to the tenets of the Helsinki Declaration. Informed consent was obtained from patients or their parents after they received a detailed explanation of the study. The study included 35 boys and 7 girls, 6–17 years of age, who had visited Aravind Eye Hospital in Madurai during March 2010–February 2013 with a history of redness, itching, and swelling of the eye. Of the 42 children, 28 had anterior chamber granuloma (≈2–3 mm diameter); 14 had subconjunctival granuloma (≈5 mm diameter). The patients were from 33 different villages in the states of Tamil Nadu and Kerala in South India. 

All patients underwent a complete ocular examination with a slit lamp and indirect ophthalmoscopy, and each also had a complete physical examination. Common causes of eye disease, such as tuberculosis, sarcoidosis, and fungal granuloma, were ruled out by clinical, radiologic, serologic, and histopathologic examinations. Patients’ granuloma samples were tested with nested PCR that targeted the MPB64 and 28S rDNA genes to rule out the possibility of tuberculosis and fungal infections, respectively. All 42 granuloma samples were subjected to assays (Power SYBR Green PCR Master Mix; Applied Biosystems, Warrington, UK) targeting rDNA spanning the internal transcribed spacer (ITS) 2 sequence of the trematode with custom-designed primers. The real-time PCR–amplified products were analyzed by using bidirectional sequencing and BLAST analysis to identify the trematode at the species level.

### Excision of the Granuloma and DNA Isolation

Twenty-eight anterior chamber granulomas were aspirated from children under general anesthesia by using aseptic precautions and a 25-gauge needle passed through the limbus (*18*). Fourteen subconjunctival granulomas were surgically excised from children under general anesthesia. Samples were stored at –80°C. Total genomic DNA was extracted and purified from the 42 biopsied specimens by using the QIAamp DNA Mini Kit (QIAGEN, Hilden, Germany), according to the manufacturer’s instructions (*28*). The granulomas were first immersed in 180 µL of ATL buffer (QIAGEN) with 20 µL proteinase K at 56°C for 2 h. Granulomatous tissue was lysed by using the QIAamp DNA Mini Kit tissue lysis procedure. DNA was extracted with a final elution volume of 100 µL and stored at –20°C.

### Real-Time PCR Assay

The rDNA sequence spanning the ITS2 region was amplified from DNA obtained from the biopsied specimens. Real-time PCR was performed according to standard protocol (*25*–*27*). To identify the trematode by real-time PCR, a new set of primers was designed in our laboratory on the basis of conserved ITS2 sequences of the digenean trematodes, including *P. varium*, *P. cheni*, *Haplorchis* *pumilio*, and others belonging to the Heterophyidae sequences available in GenBank (Table 1). Real-time PCR was conducted in a 25-µL reaction mixture containing 12.5 µL (× 2) of Power SYBR Green PCR Master Mix solution, 2.0 µL of double-distilled DNase-free water (Affymetrix, Cleveland, OH, USA), 100 nmol/L of each primer, and 10 µL of the DNA extracted from each granuloma sample. PCR was performed on an ABI 7900HT Fast Real-Time PCR System (Applied Biosystems) with the following cycling conditions: 50°C at 2 min, 95°C for 10 min, and 40 cycles of amplification (95°C for 15 s and 60°C for 1 min). No template controls (i.e., nuclease-free water) were included. Quantitative standards (i.e., DNA of *F. gigantica* recovered from cow liver) were included each time, and PCR was undertaken to detect false-positive results that could occur because of contamination and to construct a standard curve. Products amplified with real-time PCR were further analyzed by agarose gel electrophoresis. All assays were performed in triplicate.

**Table 1:** Primers used for amplification of the DNA region ITS2 and the 28S rDNA of trematode found as the source of ocular granulomas in children, South India*

DNA partition	Primer name	Primer sequence, 5′→ 3′	DNA amplicon size, bp	Application	Reference
ITS2	3SF	GGTACCGGTGGATCACTCGGCTCGTG	539	PCR and sequencing	(*29*)
	BD2R	GGGATCCTGGTTAGTTTCTTTTCCTCCGC			
ITS2	AP101F	ATGAATGGCGCAGCTTTGACATCG	156	Real-time PCR	This study
	AP101R	AAAGCACAAGGAAATCACGCCAGC			
ITS2	AP102 F	AGAGCGCAGCCAACTGTGTGA	369	Real-time PCR and sequencing	This study
	AP102 R	TGCCACGTCCTAGCATCAGCC			
28S rDNA	AP103 F	AGAGCGCAGCCAACTGTGTGA	715	PCR and sequencing	This study
	AP103 R	TGCCACGTCCTAGCATCAGCC			

*ITS, internal transcribed spacer.

### DNA Sequencing and BLAST Analysis

The real-time PCR amplicons were analyzed on 2% agarose gel stained with ethidium bromide. Confirmed samples were further subjected to bidirectional sequencing. The amplified products were loaded on the gel and purified by using the DNA Purification Kit (Promega, Madison, WI, USA). The cyclic sequencing reaction was performed with Big-Dye Terminator v3.1 Cycle Sequencing Kit (Applied Biosystems). Samples were denatured at 96°C for 2 min and then cycled 28 times at 96°C for 10 s, 52°C for 10 s, and 60°C for 4 min. Unincorporated nucleotides were removed by using sodium carbonate, 125 mmol/L EDTA, and absolute alcohol. HI-Di Formamide (Applied Biosystems) was used to stabilize the single-strand template before sequencing was performed in the ABI 3130 Genetic Analyzer (Applied Biosystems). Sequences were analyzed by using BLAST for species-level identification.

### Environmental Sample Analysis

#### Sampling of Snails and Harvesting of Trematode Cercariae

**Figure 1:**
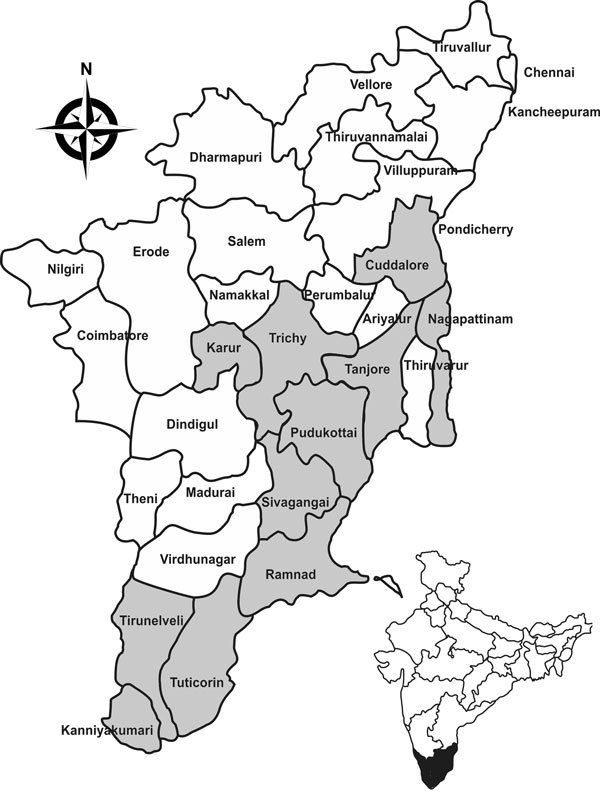
Eleven district sites (gray shading) where snails were collected in the state of Tamil Nadu, India, for testing as part of a study of ocular granulomas in children. Inset shows location of Tamil Nadu in India.

Village ponds and rivers were selected on the basis of patients’ history of frequent bathing in them before symptoms developed. Village maps were obtained from village authorities, and ponds and rivers were surveyed for snail collection. We visited 68 villages in 11 districts in Tamil Nadu to collect snails (Figure 1) during a 3-year period (March 2011–February 2014).

Snails were collected by hand picking them from the muddy ponds and rivers of the disease-affected localities. We collected ≈7,200 snails comprising 7 species. Each site was sampled twice during the study period. Snails were placed in plastic containers filled with water from the same habitat and transported alive to the laboratory. The snails were cleaned by using pond water to reduce the debris and placed separately in a small plastic petri dish containing 50 mL of filtered pond water. The snails were exposed to sunlight for 1.5–2 h to induce shedding of cercariae. Each snail was carefully observed under a dissecting microscope every day after the exposure of sunlight; when the shedding was complete, the cercariae were separated. The snails were maintained in the laboratory at room temperature for up to 2 weeks. The filtered pond water was replenished daily to avoid pH changes (*30*–*32*). 

The procedure was repeated until several cercariae were obtained from each species of snail. The released cercariae were collected individually and placed in 95% ethanol for DNA extraction and 10% formalin for morphologic identification by using a borax-carmine procedure, as described (*33*). After complete cercarial examination, snails were treated with 10% sodium hypochlorite and dried in sunlight. The dry shells of snails were sent to the Zoological Survey of India in Kolkata for species identification.

#### DNA Isolation from Trematode Cercariae

Cercariae preserved in 95% ethanol were centrifuged at 12,000 rpm for 10 min, after which the supernatant was discarded and pellets were air-dried. Genomic DNA extraction from cercariae was performed by using the QIAamp DNA Mini Kit, according to the manufacturer’s instructions (*28*). DNA was eluted with 100 µL of buffer AE from the DNeasy spin column (QIAGEN). The DNA concentration was measured by using the NanoDrop spectrophotometer (Thermo Fisher Scientific, Grand Island, NY, USA) and was stored at –20°C.

#### PCR Assay

Unlike the clinical samples, which used real-time PCR to increase sensitivity because of limited amounts of DNA, conventional PCR was used for the environmental samples because of availability of adequate amounts of DNA from freshwater snails. The ITS2 and 28S rDNA regions were amplified from the trematode cercariae DNA by using standard protocol (*29**,**34*–*36*). For species-level identification of the trematode, we used universal primers created on the basis of conserved ITS sequences of the *Schistosoma* species of trematodes (*29*)*.* We also used custom-designed primers targeting the conserved ITS2 and 28S rDNA sequence of the digenean trematodes, including *P. varium*, *P. cheni*, *H. pumilio*, and others belonging to the family Heterophyidae (reported in GenBank; Table 1). PCR was carried out in a 20-µL reaction mixture containing 10X PCR buffer, 10 mmol/L deoxynucleotide triphosphates, 25 mmol/L magnesium chloride, 3 U/µL Taq DNA polymerase (these 4 reagents from Bangalore Genei, Bengaluru, India), 1X Q-solution (Qiagen), 7 pmol of each primer, and 5 µL of DNA template (cercariae DNA). PCR conditions for ITS2 (Thermal Cycler PTC-200; Bio-Rad, Hercules, CA, USA) were as follows: 5 min at 94°C for initial denaturation, followed by 35 cycles (30 s at 94°C for denaturation, 38 s at 57°C for primer annealing, and 72°C for 42 s for extension) for adequate amplification, and a final extension at 72°C for 7 min. The PCR conditions for 28S rDNA were similar to ITS2 except that we used 56°C for primer annealing and 1 min for the initial extension. After amplification, electrophoretic separation of PCR products was performed on 1.5% agarose gel prestained with ethidium bromide and visualized by ultraviolet illumination.

### Molecular Sequencing of Environmental Trematode DNA

The amplified PCR products from the DNA extracted from the environmental trematode cercariae were subjected to bidirectional sequencing in the ABI 3130 Genetic Analyzer, and the sequences were deposited in GenBank (accession nos. KM226892–KM226899). BLAST analysis of the sequences was performed for species-level identification.

## Results

### Patient Demographics and Clinical Findings

**Figure 2:**
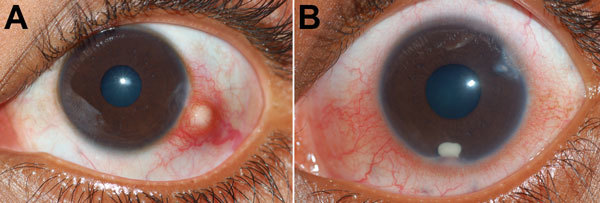
Clinical photographs of patients’ eyes in study of ocular granulomas in children, South India. A) Left eye of a 14-year-old boy with a distinct subconjunctival granuloma; B) left eye of a 7-year-old boy with distinct grayish-white granuloma in the eye’s anterior chamber.

Forty-two children with ocular granulomatous inflammation who sought care at our hospital were all exposed to pond or river water. None had a history of consumption of raw or undercooked fish. Fourteen of the 42 children had subconjunctival granuloma (Figure 2, panel A); 28 had anterior chamber granuloma (Figure 2, panel B). Results of general physical examinations were unremarkable. None of the patients had chronic systemic granulomatous disease, which is known to be associated with uveitis and includes tuberculosis and sarcoidosis.

### Molecular Analysis of the Patient Samples

**Figure 3:**
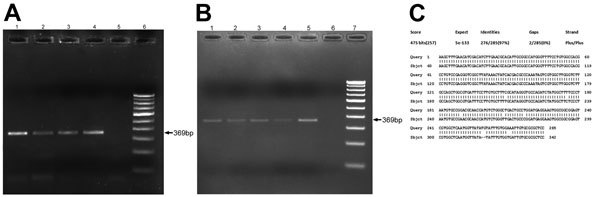
Real-time PCR amplification of ocular granuloma DNA obtained from patients infected with trematodes, South India. Gel electrophoresis was performed on 2% agarose gel by using Power SYBR Green Real-Time PCR (Applied Biosystems, Warrington, UK). A) Lanes 1–4 show subconjunctival granuloma DNA; lane 5, negative control; lane 6, 100-bp DNA marker. Arrow indicates 369-bp amplified DNA product. B) Lanes 1–5 show anterior chamber granuloma DNA; lane 6, negative control; lane 7, 100-bp DNA marker. Arrow indicates 369-bp amplified DNA product. C) BLAST (http://blast.ncbi.nlm.nih.gov/Blast.cgi) analysis output of patient granuloma DNA sequence showing maximum identity with internal transcribed spacer 2 region gene sequence of the *Procerovum* species resembling GenBank reported sequence EU826639.1 from Vietnam.

Results of testing for 13 of the 42 granuloma samples analyzed were positive for trematode DNA by using SYBR Green quantitative real-time PCR. Of the 13 patients with positive samples (Table 2), 8 had subconjunctival granulomas, and 5 had anterior chamber granulomas. Real-time PCR was performed on 2% agarose gel, and BLAST analysis of amplified sequences revealed the closest identity with the trematode *Procerovum* spp. (family Heterophyidae) (GenBank accession no. KM226891; Figure 3, panels A–C). All samples were found by nested PCR to be negative for *Mycobacterium* *tuberculosis* and fungal infection.

**Table 2:** Summary of clinical features of 13 patients whose samples were positive for trematode DNA in study of ocular granulomas in children, South India*

Sample no.	Type of granuloma	Patient age, y/sex	Duration of granuloma, ≈d
1	SCG	9/M	30
2	ACG	9/F	60
3	ACG	11/M	30
4	SCG	11/F	7
5	SCG	15/M	35
6	ACG	9/M	60
7	SCG	11/M	30
8	SCG	11/M	15
9	SCG	18/M	30
10	ACG	7/M	90
11	ACG	14/M	15
12	SCG	6/M	15
13	SCG	16/M	10
*Testing performed with Power SYBR Green Real-Time PCR (Applied Biosystems, Warrington, UK). SCG, subconjunctival granuloma; ACG, anterior chamber granuloma. All patients reported bathing in a pond or river, and all trematodes were determined to be *Procerovum* spp.			

### Environmental Sample Analysis

**Figure 4:**
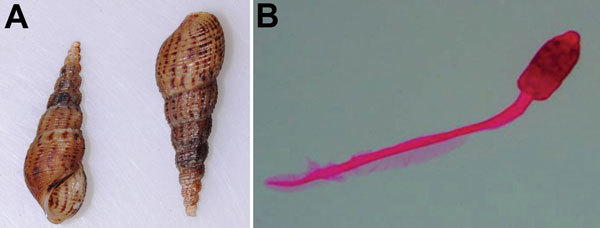
Snail and trematode cercaria from study of ocular inflammation in children, South India. A) *Melanoides* *tuberculata* snails collected from a pond that was the focus of the infection. B) Staining and light microscopy image of the cercaria larval stage recovered from the snails (original magnification ×200).

**Figure 5:**
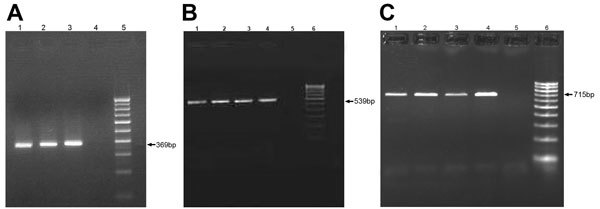
PCR amplification of trematode cercaria DNA obtained from *Melanoides* *tuberculata* snails in study of ocular inflammation in children, South India. Gel electrophoresis was performed on 1.5% agarose gel. A) Internal transcribed spacer 2 region; arrow indicates 369-bp amplified DNA product. Lanes 1–3, trematode cercariae DNA; lane 4, negative control; lane 5, 100-bp DNA marker. B) Internal transcribed spacer 2 region; arrow indicates 539-bp–amplified DNA product. Lanes 1–4, trematode cercariae DNA; lane 5, negative control; lane 6, 100-bp DNA marker. C) 28S rDNA region; arrow indicates 715-bp amplified DNA product. Lanes 1–4, trematode cercariae DNA; lane 5, negative control; lane 6, 100-bp DNA marker.

**Figure 6:**
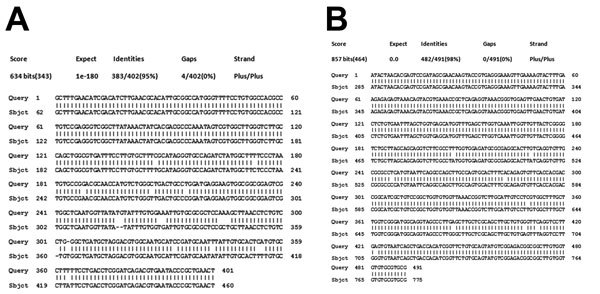
BLAST (http://blast.ncbi.nlm.nih.gov/Blast.cgi) analysis output of environmental trematode cercaria DNA sequences from South India. A) Internal transcribed spacer 2 DNA sequence shows maximum identity with *Procerovum* species and resembles GenBank reported sequence EU826639.1 from Vietnam. B) 28S rDNA sequence shows maximum identity with *Procerovum* *varium* and resembles GenBank reported sequence HM004184.1 from Thailand.

A total of 7 species of snails were collected from 68 village ponds and rivers. On the basis of shell morphology, snails were identified by the Zoological Survey of India as *Bellamya* *dissimilis, Pila virens, Melanoides* *tuberculata, Lamellidens* *marginalis*, *Paludomus* *transchauricus*, *Indoplanorbis* *exustus*, and *Thira scraba*. Among these snail species, only *M. tuberculata, I. exustus*, and *T. scraba* were found to be infected with trematode larvae and released cercariae in the laboratory, although all snail types were exposed to the same environmental conditions. The snail *M. tuberculata* was found in 57 of the 68 water bodies surveyed and comprised on average 20% (range 0%–40%) of all snails collected from the sites. *M. tuberculata* snails released the trematode cercaria identified as *P. varium* (Figure 4, panels A, B). Figure 5 (panels A–C) shows the PCR-amplified product of trematode cercaria DNA that was performed on 1.5% agarose gel. Molecular sequencing and BLAST analysis of the PCR amplicon sequence confirmed maximum sequence similarity with *P. varium* (GenBank accession nos. KM226892 and KM226894) (Figure 6, panels A, B). Besides releasing *P. varium,* the *M. tuberculata* snails also released cercariae of 3 other species, represented by *Haplorchis* *pumilio* (GenBank accession no. KM226895; family Heterophyidae), *Giganthobilharzia* *melanoidis* (GenBank accession no. KM226896; family Schistosomatidae), and an unidentified species of the Renicolidae family (GenBank accession no. KM226897). Cercariae released by snails of the other 2 species, *T. scraba and I. exustus*, were identified as *Acanthostomum* *burminis* (GenBank accession no. KM226898) and *Isthmiophora* *hortensis* (GenBank accession no. KM226899), respectively.

## Discussion

In this study, pediatric granulomatous eye disease developed in a group of 42 children with a history of exposure to village pond and river water in sites we surveyed in South India. Common causes of granulomas (e.g., tuberculosis, sarcoidosis, and fungi) were ruled out by various diagnostic (i.e., clinical, radiologic, serologic, and histopathologic) techniques. Our study results, determined by using molecular techniques, confirmed that the cercaria stage of a digenetic trematode, *P. varium* (family Heterophyidae), was implicated in human granuloma tissue. The cercariae are environmental pollutants in snail-infested waters.

Heterophyid flukes, including *Procerovum* spp., need 1 definitive host and 2 intermediate hosts to complete their life cycle. The fluke releases embryonated eggs in the host’s feces. The cercaria larvae develop in snails, the first intermediate host; the cercariae encyst as metacercariae in the tissues of a suitable fish, which is the second intermediate host for the parasite. The definitive host is infected by ingesting raw or undercooked fish containing metacercariae; after ingestion, the metacercariae excyst (i.e., attach to the mucosa of the small intestine) and mature into adults. Haplorchid metacercariae were abundantly reported in many freshwater fish in Taiwan and caused cercarial infection in the eye of eel; histopathologic sections showed numerous metacercariae in the muscle tissues, subcutaneous tissue, and cartilage, and edema and hemorrhage were seen in the eye (*20**,**22*).

In India, heavy infections of metacercariae were reported in the freshwater fish *O. melastigma* (*21*). Morphologically, these metacercariae were identified as the cysted stage of *P. varium*. Natural infections of *P. varium* were found in birds (e.g., the pond heron *A. grayii*) in the same geographic area. Adult flukes of these parasites were successfully raised from metacercariae in chicks, ducklings, and mice. Laboratory and field studies confirmed that the snail *Thiara* *tuberculata* acts as the first intermediate host (*21*).

In our environmental analysis, of 7 species of snails identified, *M. tuberculata* and 2 other species released cercariae in the laboratory. *M*. *tuberculata* snails were found predominantly in most village ponds and rivers tested. The DNA sequence of the cercarial larva isolated from the snail was identical to that of *P. varium* seen in the patients’ granuloma samples. Besides *P. varium, M. tuberculata* snails also released cercariae of 3 other trematode species: *H. pumilio, Giganthobilharzia* *melanoidis*, and a representative of the family Renicolidae. *Haplorchis* is a fish-borne intestinal fluke that is highly prevalent in Southeast Asia, but little is known about the infection dynamics and clinical symptoms in hosts, including humans. However, none of these 3 cercarial types were found in our patients’ ocular granulomas.

In our patients’ sample analysis, 13 (31%) of 42 samples were positive for *P. varium*. Why the remaining patients’ samples were negative for trematode DNA is unclear. It is possible that the parasite structures disintegrated rapidly as a result of the localized intense inflammatory response of the host or because of necrosis. Alternatively, different species could have caused the illness. When tested by nested PCR, all samples were negative for *Mycobacterium tuberculosis* and fungus. Potentially blinding granulomatous eye disease in children was previously misdiagnosed as tuberculosis; for several decades, these children were receiving antituberculosis treatment, which was ineffective in controlling the inflammation (*37*). In Brazil, similar types of ocular infection were reported as adiaspiromycosis caused by nonbudding, thick-walled adiaconidia of the Emmonsia spp. fungus (*38*). However, none of our samples showed evidence of fungus or tuberculosis by histopathology or molecular methods.

Although parasitic diseases are a major public health problem in developing countries, they are grouped under “neglected tropical diseases” (*39*). We isolated 5 more trematode species from snails in the same region in addition to *P. varium*, but little is known about the infection dynamics and clinical symptoms in their hosts. Further research is needed to understand the prevalence of various trematode-borne diseases, including ocular parasitosis, in South India.
